# Interdisciplinary teaching in family medicine teaching units: the residents’ points of view

**Published:** 2018-07-27

**Authors:** Louis-François Dallaire, Caroline Rhéaume, Lucie Vézina

**Affiliations:** 1 Department of Family Medicine and Emergency Medicine, Faculty of Medicine, Université Laval, Québec, Canada; 2 Centre de recherché de l’Institut universitaire de cardiologie et de pneumologie de Québec, Québec, Canada

## Abstract

**Background:**

Interdisciplinary teaching (IDT) is the norm in Canadian family medicine residency programs. Literature on IDT reports many academic, collaborative and organizational benefits, but little is known about family medicine residents’ own perspectives of IDT. The purpose of this study was to explore family medicine residents’ points of view on IDT in family medicine teaching units (FMTU).

**Methods:**

A mixed methods design combined interviews and self-completed online questionnaires to explore participants’ perceptions of IDT during residency. Content analysis was conducted on the qualitative data and univariate analysis statistical tests on means and proportions were conducted on the quantitative survey questions.

**Results:**

A total of 125 family medicine residents from 12 FMTU affiliated with Université Laval (Quebec City) participated in the study (11 interviews and 114 online questionnaires). Participants perceived significant benefits of IDT, including clinical knowledge, complementary perspectives and interprofessional collaboration skills. However, they believe that IDT works best when the educators adapt their teaching to the specific needs of residents in family medicine.

**Conclusion:**

These findings support those of previous IDT research and highlight the positive impacts of interdisciplinary education in family medicine residency, especially on interprofessional collaboration. IDT should remain an essential component of the family medicine curricula.

## Abstract (fr)

**Contexte:**

L’enseignement interdisciplinaire (EID) constitue une norme dans les programmes canadiens de résidence en médecine familiale. La littérature disponible sur l’EID fait état de plusieurs bénéfices académiques, collaboratifs et organisationnels, mais elle rend peu compte des points de vue des résidents sur ce type d’enseignement. Cette étude a pour objectif d’explorer les points de vue des résidents en médecine familiale quant à l’EID offert dans les unités de médecine familiale (UMF).

**Méthodes:**

Un devis mixte a été utilisé, s’appuyant sur des entrevues semi-dirigées et des questionnaires auto-administrés en ligne. Une analyse de contenu a été effectuée pour le volet qualitatif, et des analyses univariées et bi-variées ont été réalisées pour les données obtenues dans les questionnaires auto-administrés.

**Résultats:**

Un total de 125 résidents en médecine familiale, rattachés aux 12 UMF relevant de l’Université Laval (Québec), a participé à l’étude (11 entrevues et 114 questionnaires en ligne). Les participants reconnaissent des bénéfices significatifs à l’EID, tels l’acquisition et l’approfondissement de connaissances cliniques essentielles, l’intégration de perspectives complémentaires sur les problèmes de santé, et le développement d’habiletés à la collaboration interprofessionnelle. Ils estiment toutefois que l’EID peut s’optimiser en s’adaptant davantage aux besoins spécifiques des résidents en médecine familiale.

**Conclusions:**

À l’instar des études antérieures, les résultats de cette recherche mettent en relief les impacts positifs de l’EID pendant la résidence en médecine familiale, particulièrement ceux qui sont liés à l’apprentissage de la collaboration interprofessionnelle. L’EID devrait par conséquent demeurer une caractéristique essentielle des programmes de résidence en médecine familiale.

## Introduction

For decades, in Canada and other countries, health professional educators (HPE) from allied health disciplines (e.g., nursing, social sciences, psychology, kinesiology and pharmacy) have been teaching future family physicians, both in academic and clinical settings such as family medicine teaching units (FMTU). Interdisciplinary teaching (IDT) is defined as the use of methods and analytical frameworks from more than one academic discipline, with the goal of providing a more complete and coherent framework of analysis. IDT differs from interprofessional education (IPE), where students from two or more professions learn together to improve collaboration and health outcomes.^1^ IDT is the norm in Canadian family medicine education and is the joint responsibility of the teaching faculty and the clinical setting, such as the hospital or health and social services centre where medical students complete their residency.^2^

Although the literature on IDT in medicine is somewhat limited, it reports many benefits. From an academic perspective, IDT contributes to the learning of essential medical knowledge and skills,^3-6^ the integration of a patient-centred approach,^7^ reflexivity,^8^ and a higher tolerance for uncertainty,^5^ while developing communication skills,^7-9^ problem-solving strategies,^5^ and patient education methods.^6^ By giving medical students a better understanding of the roles of the various professions and a more positive perception of their contributions, IDT is also believed to have a positive impact on the development of interprofessional collaboration skills.^10-13^ These benefits are coherent with the evaluation training objectives in family medicine (as defined by the College of Family Physicians of Canada) and with current expectations that health services should revolve around interprofessional collaboration.^14,15^ Finally, given the fact that doctors earn considerably more than the other health professionals, IDT is considered a cost-effective teaching approach, especially in clinical settings where doctors often lack the time and resources to fully invest themselves in teaching.^1,4,13,16,17^

While these studies highlight the positive impacts of IDT in medical education, not many have documented the views of the family medicine residents themselves. The few available studies that have done so were either restricted to the contribution of educators from a specific profession (such as nurses^16^ or pharmacists^3^) or were conducted in non-clinical contexts.^12^ Given the significant investment in HPE for teaching in family medicine residency, it seemed fitting to explore the residents’ points of view on the IDT educational model. Since family medicine residents experience IDT on a daily basis, we believe this could provide a valuable description and understanding of its contribution to family medicine residency. The goals of this study were to explore and describe family medicine residents’ experiences with IDT and to develop recommendations to Canadian family medicine departments wishing to make the best use of their HPE.

## Methods

### Study design

An exploratory study was conducted between May 2015 and January 2016, based on a sequential mixed method design. Using both qualitative and quantitative data is considered effective for presenting a complete description of a phenomenon,^18^ and so this seemed appropriate given the goals of this study and the limited literature available on IDT in medicine. The first sequence of data collection was conducted through individual interviews to gain a deeper understanding of participants’ experiences with IDT. The interviews lasted approximately 30 minutes, were based on a semi-structured interview guide and were conducted by a research professional. The outcomes measured were participants’ experiences of IDT during family medicine residency, their overall assessment of this teaching approach and their recommendations for better use of HPE during residency. Interviewees were asked to illustrate their experience of IDT with specific examples; for instance, they were asked describe one positive and one negative experience of IDT during their family medicine residency. All interviews were recorded with the agreement of participants in order to facilitate transcription.

The second sequence of data collection was conducted through anonymous self-administered online questionnaires using Lime Survey software. Participants were questioned about the interdisciplinary teaching team at their residency site, about the ways they benefitted from IDT during their family medicine residency, and about the level of motivation they felt towards being taught by HPE. The online questionnaire featured 21 items based on the benefits of IDT, as selected from the literature and divided into four categories (*learning, complementarity, reflexivity*, and *interprofessional collaboration*). Participants were asked to indicate their level of agreement with each item on a five-point Likert scale, with scoring ranging from “totally disagree (1)” to “very strongly agree (5).” An initial version of the online questionnaire was developed and pre-tested by the research team; later on, we slightly modified this first draft to explore additional concepts raised by interviewees. The final version of the questionnaire could be completed in 15 minutes. Three reminders were addressed to non-respondents to stimulate their participation in the study.

### Participants and settings

All of the family medicine residents (n=233) in the 12 FMTU linked to the Department of Family & Emergency Medicine (DFEM) at Université Laval (Quebec City, Canada) were solicited via email about the goals and participation requirements of the study. While all the residents were eligible to complete the online questionnaire, only second-year and third-year residents were asked to participate in the interviews, in order to better reflect the points of view of residents with extended experiences of IDT. To diversify the sources of data and to take local realities into account, attempts were made to ensure representation of both urban and semi-urban FMTU in the sample since urban FMTU typically have more residents than semi-urban FMTU.

For the interviews, we recruited four residents in semi-urban FMTU (Gaspé, Lanaudière, and Lévis) and seven from urban FMTU. Saturation of data was quickly reached after these 11 interviews, signalling that no further interviews were necessary.^19^ Interviews were either conducted at the resident’s FMTU or by phone. For the survey, we recruited 114 residents from all 12 Université Laval FMTU (107 of whom completed the online questionnaire) by writing emails sent from the Family Medicine Department and sending follow-up reminders. A link to the survey was included in all those emails.

### Data analysis

A five-step content analysis^20^ of the qualitative data was conducted by the first author using Provalis QDA Miner qualitative analysis software. Interviews were transcribed and then pre-analyzed to gain a global perspective of the residents’ points of view. Data were then classified according to categories emerging from the available literature on IDT. Following that step, zones of convergence and divergence were identified, and the resulting analysis was validated by a subgroup of residents to ensure proper representation of the participants’ points of view. The online questionnaire was then modified as a result of the interview findings; for instance, the survey asked participants about their initial reaction to being taught by HPE, and about the conditions that most favored interest and motivation in IDT.

Quantitative data analysis was conducted using SAS software (SAS Institute Inc., Cary, NC). Internal consistency tests (Cronbach alphas) were performed on the main concepts and univariate analysis was conducted to describe all variables. Qualitative and quantitative analyses were then compared to seek areas of convergence and divergence, with the goal of producing a comprehensive and representative view of the participants’ experiences with IDT.

### Ethical considerations

The study was approved by the research ethics committee of Université Laval, Quebec. Participants were handed a consent form detailing the nature and goals of the study, explanations of what was expected of them, the measures taken by the research team to ensure anonymity and the potential risks of participating in such a study.

## Results

A total of 125 residents participated in the research, representing a satisfactory participation rate of 53.6%. The online questionnaire was completed by 114 residents. The completed questionnaires were filled in online by participants in their location of choice. Table 1 presents characteristics of both samples.

**Table 1:** Samples’ characteristics

Characteristics	Interview (n=11)	Online questionnaire (n=114)
Gender	Female : 91.0 % (10) Male : 9.0 % (1)	Female : 71.3 % (82) Male : 28.7% (32)
Age (mean)	27.5	29.9
Previously studied and practiced medicine outside of Canada	9.0 (1)	16.7 % (19)
Previously completed nonmedical studies	36.4 % (4)	11.4 % (13)
Urban FMTU	81.8 % (9)	64.9 % (74)
Semi-urban FMTU	18.2 % (2)	35.1 % (40)

### Description of participants’ experiences with IDT in a FMTU

Participants’ viewpoints illustrate the diversity of HPE teaching in FMTU. The HPE most frequently mentioned as being involved in teaching were social workers (83.3%; n=95), pharmacists (79%; n=09) clinical nurses (78.1%; n=89), and psychologists (72.8%; n=83). While the teaching contributions of other HPE (kinesiologists, dieticians, physiotherapists, research professionals) were also praised by many of the interview participants, they were mentioned less frequently as fewer of these HPE teach in Quebec FMTU. “The physiotherapist gave us a seminar on positional plagiocephaly, which we hadn’t been taught much about in med school, and it was very helpful because [family physicians] have a part to play in detecting it and in referral to the appropriate specialists” (p2).

Participants widely acknowledged the positive contribution of HPE during family medicine residency, with a global appraisal score of 4.1 out of 5. They considered that HPE were actively involved in their teaching (4.2/5), competent in that role (4.3/5) and involved in a wide variety of teaching activities such as non-clinical teaching (97.4%), interprofessional education (84.2%), direct supervision (96.5%), and case discussion supervision (51.7%). Most participants also considered collaborative care with HPE as a particularly fruitful learning opportunity: “In the early stages of my residency, there was this elderly diabetic patient whose medication just wasn’t working, and I couldn’t figure out what was wrong. The pharmacist helped me adjust his medication and sat down with me to assess my difficulties in this area and to provide me with the information that I lacked” (p8).

HPE often co-teach with family doctors, most frequently in direct supervision and non-clinical teaching. Although participants appreciated this complementary approach, only 12.4% (n=13) of them considered the presence of a doctor to be essential in these specific activities. “Well, obviously, the social worker won’t give us a seminar on diabetes! But if we’re having a class on mental-health related issues, he’s as good as any doctor” (p6). This trusting relationship was based upon participants’ core belief that HPE have a high level of professional expertise in their own disciplines, a belief that comes with high expectations: “They [*HPE*] are experts in their field, so when they’re teaching us something, I expect to get something more out of it than if a doctor was teaching the same topic” (p9).

### IDT-related benefits for residents

The results indicated that participants considered IDT overall as a positive element of family medicine residency (Table 2). The contribution of HPE was especially valued for their teaching of interprofessional collaboration skills but also for the complementarity of perspectives from a wide variety of disciplines.

**Table 2:** Participants’ perception of IDT-related benefits

(N=107) Scale of 1 to 5	Mean	Standard deviation
**LEARNING**	**3.8**	**± 0.7 3,9 (median)**
1. Learning technical skills	3.4	± 1.2
2. Integration of differential diagnosis process	3.5	± 1.0
3. Knowledge/information on health issues	4.0	± 0.8
4. Supportive psychotherapy	4.0	± 0.9
5. Physical examination of patients	3.2	± 1.2
6. Mental examination of patients	3.5	± 1.0
7. Patient-centered communication skills	4.2	± 0.8
8. Patient-centered negotiation skills	4.1	± 0.8
9. Information on treatment options	4.2	± 0.8
**COMPLEMENTARITY**	**4.1**	**± 0.7 4,0 (median)**
10. Consolidation of previously acquired knowledge	4.1	± 0.8
11. Additional perspectives on health issues	4.2	± 0.8
12. Identification of a wider range of treatment options	4.2	± 0.8
**REFLEXIVITY**	**3.9**	**± 0.7 4,0 (median)**
13. Reflexivity on patient-doctor relationship issues	4.0	± 0.8
14. Higher level of comfort with complex clinical situations	3.9	± 0.8
15. Development/integration of reflexive practice skills	3.9	± 0.8
16. Higher level of self-confidence	3.7	± 0.9
17. Constructive feedback on knowledge and skills	3.9	± 0.9
**INTERPROFESSIONAL COLLABORATIONS**	**4.3**	**± 0.7 4,3 (median)**
18. Understanding other health professionals’ roles	4.2	± 0.8
19. More positive perception of other health professionals	4.3	± 0.7
20. Development of collaborative care skills	4.3	± 0.8
21. Being able to direct patients efficiently through the healthcare system	4.3	± 0.7
**OVERALL APPRECIATION (global score)**	**4.0**	**± 0.7 4,0 (median)**
Quantitative data analysis was conducted using SAS software (SAS Institute Inc., Cary, NC). Statistical tests on means and proportions (t-test, χ^2^) were conducted.		

We also asked participants to select the three main benefits of IDT. “Understanding other health professionals’ roles” and “Being able to direct patients efficiently through the healthcare system” were tied in first place (17%; n=18), with “Development of collaborative care skills” coming second (14%; n=15) and “Identification of a wider range of treatment options” in third place (9.5%; n=10). These results highlight the positive impact of IDT on training for interprofessional collaboration, since the two top choices were related to this concept. Participants felt that being taught by HPE allowed them to go beyond the theoretical concepts of interprofessional collaboration and to actually experience it: “[IDT] enables us to become familiar with the work of other health professionals, to know exactly when and how they can be helpful to our patients, because the point isn’t to simply dump our patients on these professionals’ shoulders – the point is to learn to work efficiently as a team, so that each of us can actually do what we’re supposed to do with every patient. This is something we experimented in our day to day contacts with [HPE]” (p7).

### Participants’ recommendations about IDT

Participants were also asked about how IDT could be improved during residency, and about their recommendations to family medicine residency programs. Their first recommendation was to maintain the involvement of HPE in teaching. When asked if their initial (pre-residency) perception of IDT was positive, negative or neutral, and how this perception had evolved through their residency, 52.4% (n=55) of the participants mentioned that their initial perception of IDT was positive and that their experience met their expectations; and 42.9% (n=45) reported that while their initial perception was positive, their experience had actually surpassed their expectations. Consequently, they considered that IDT should remain a key feature in FMTU, and some participants even said that the loss of IDT during residency would be detrimental to family medicine residents:

“The relevance of HPE teaching to family medicine residents shouldn’t be questioned, because their expertise provides us with additional knowledge, some of which cannot be taught as effectively by doctors” (p1). Many of the interviewees voiced a critical concern that IDT might be cut from family medicine residency given the health system reform currently happening in the province of Quebec: “It would be a shame for us to lose such a high standard of education, just because [our government] doesn’t want to support IDT anymore” (p8).

This being said, participants observed that certain conditions must be present for IDT to achieve its full potential. The most frequently expressed criticism (60%; n=63) was that HPE sometimes overlook that they are teaching family physicians – and not, at this participant pointed out, students in their own professional field: “When [*the dietitian*] taught us how to calculate the energy requirements of our patients, I didn’t find it too useful. It felt like something that was closer to her professional role than to ours” (p4).

The second most frequently expressed criticism (53.3%; n=56) was that HPE sometimes repeat the teaching of skills the residents feel they have already acquired. “There are things we’re supposed to know at the beginning of residency. If you still have no clue about the ways to establish a good doctor-patient relationship, the solution isn’t to have more classes on this topic” (p5). And although 43.8% (n=46) of the participants expect HPE to be experts in their own profession, that expertise can be a double-edged sword since residents are sometimes apprehensive about what is expected of them. “At first, I was a bit wary about having a social worker or a psychologist observing me with actual patients. I don’t have their level of expertise about establishing a helpful professional relationship with patients, so I was scared of being judged upon criteria that I couldn’t live up to” (p1).

## Discussion

This study provides an original contribution to knowledge about IDT in medical education by focusing on the points of view of those who experience it and who should be considered key informants about its value.

Our findings support those of previous research on IDT, especially studies illustrating the development and integration of useful knowledge and skills,^1,3-5,7,8,17^ the identification of a wider range of solutions to the more complex clinical situations^1-3^ and the benefits of IDT on training for interprofessional collaboration.^3,4,8,10,12,13,17^ This study also supports and expands the understanding of IDT’s organizational benefits.^1,4,13,16,17^ Participants’ points of view also highlight that while HPE obviously can’t (and shouldn’t) replace doctors in medical education, they are quite able independently to handle the teaching relevant to their own professional expertise. Although the presence of HPE in FMTU is often funded by healthcare organizations, this investment actually frees doctors from the teaching tasks for which their presence isn’t essential, allowing them to devote more time to their patients. This study invites healthcare organizations to recognize the distinct status of FMTU-based HPE and to support their contribution to teaching activities during family medicine residency.

Participants’ viewpoints also provided a deeper understanding of the link between IDT and interprofessional collaboration. While these two concepts are distinct – IDT being an educational model involving teachers from various health disciplines, and interprofessional collaboration referring to actual collaborative health care which involves at least two professionals from different disciplines –, one of the most original contributions of this study is to illustrate how closely interrelated they are. Through IDT, residents are exposed to a wide range of clinical expertise, enabling them to recognize and appreciate the expertise and contribution of HPE; this positive recognition allows residents to develop a trusting bond with non-physician health professionals, an essential condition for efficient interprofessional collaboration. This study illustrates that IDT fosters interprofessional collaboration, i.e., that learning from other health professionals enhances and facilitates patient-centred collaborative care.

While the results of this study indicate a high level of appreciation of IDT, they also hint at the fact that there’s room for improvement. Participants pointed out that HPE can face certain difficulties in discriminating which part of their expertise will be beneficial to the residents, leading them to teach skills that might not be appropriate for family physicians. Residents are also likely to feel that not only are they expected to become experts in family medicine, but that they are also expected to become experts in psychology, nutrition, pharmacy and so on. This is certainly not the message that IDT should convey. Family medicine departments and FMTU directors could play a significant part in solving this difficulty by helping HPE select appropriate content and teaching objectives, giving them feedback on their teaching and allowing them to participate in the faculty’s continuing professional development activities.

Finally, from a methodological perspective, we found that a mixed methods research design was particularly appropriate in this study. The qualitative data obtained through the interviews helped to strengthen and refine the online questionnaire; in turn, the quantitative data confirmed the representativeness of the qualitative analysis. The satisfactory participation rate in the study (53.6%) also supports the reliability and veracity of the results.

### Conclusion

The goal of this study was to explore and describe family medicine residents’ views and recommendations about IDT in FMTU. The findings of this study support those of earlier research on IDT and suggest that family medicine residents experience significant benefits from being taught by HPE. Residents consider these educators as experts who fully contribute to their professional development. The residents’ recommendations for optimizing the benefits of IDT in FMTU suggest that this cost-effective teaching approach should continue to be supported by healthcare organizations.

This study has certain limitations. First, the online questionnaire was not based on a scientifically validated tool. As no validated scales were specific enough for our research questions, the survey was developed from the main concepts found in the IDT literature and refined in the light of our qualitative findings. However, internal consistency tests showed a Cronbach alpha between 0.90 and 0.96, which implies that the questionnaire was a reliable tool. Also, characteristics of the samples differed in some aspects (sex, previous studies, and/or practice of medicine outside Canada). The overall sample is still representative of the population of family medicine residents in Quebec, since most residents in Université Laval studied medicine in Canada and female residents outnumber their male counterparts. Finally, it must be remembered that our study design was exploratory, that it sought to explore family medicine residents’ subjective experiences of IDT. Therefore, results do not provide an evaluation of the impact of IDT on learning medical skills and knowledge. Measuring significant knowledge and skills gained from being taught by HPE would be an important way to further study the effectiveness of IDT in family medicine residency. Finally, this research was limited to the 12 FMTU affiliated with Université Laval. Since there are currently 17 family medicine residency programs in Canada, future research on IDT could also explore the points of view of residents from other Canadian provinces. Such efforts would provide a nationwide representation of their appraisal of IDT in family medicine.

## Appendix A Online Questionnaire

Dans le contexte de la présente étude, l’enseignement interdisciplinaire se définit comme toute activité d’enseignement en UMF relevant de la responsabilité (partielle ou totale) d’enseignants provenant d’une discipline autre que la médecine *(désignés par le terme professionnels-enseignants tout au long du questionnaire)*.

### SECTION A : CARACTÉRISTIQUES SOCIO-DÉMOGRAPHIQUES

1. Sexe
  1. Féminin
  2. Masculin
2. Âge ___________
3. Milieu de résidence :
  1. UMF des Etchemins
  2. UMF Gaspé
  3. UMF Haute-Ville
  4. UMF Laval
  5. UMF Laurier
  6. UMF Lévis
  7. UMF Maizerets
  8. UMF Manicouagan
  9. UMF Nord de Lanaudière
  10. UMF Rimouski
  11. UMF Saint-François D’Assise
  12. UMF Trois-Pistoles
4. Avez-vous effectué vos études en médecine à l’extérieur du Canada ?
5. Avez-vous pratiqué la médecine à l’extérieur du Canada ?
  1. Oui → Pendant combien d’années __________________
  2. Non
6. Avant votre résidence en médecine familiale, avez-vous amorcé ou complété une résidence dans une autre spécialisation médicale ?
  1. Oui, indiquez la spécialisation ___________________________________
  2. Non
7. Avant votre formation en médecine, avez-vous fait des études universitaires dans une autre discipline ?
  1. Oui, indiquez la discipline ___________________________________
  2. Non

### SECTION B : DESCRIPTION DE L’EXPÉRIENCE D’ENSEIGNEMENT INTERDISCIPLINAIRE

1. Outre les médecins de famille, quels types de professionnels-enseignants retrouvez-vous dans votre milieu de résidence ? ***(Vous pouvez cocher plus d’une réponse).***
  ___ Infirmières cliniciennes
  ___ Travailleuses sociales
  ___ Psychologues
  ___ Pharmaciennes
  ___ Nutritionnistes
  ___ Infirmières praticiennes en soins de première ligne (IPSPL)
  ___ Physiothérapeutes
  ___ Kinésiologues
  ___ Professionnel de recherche
2. Parmi ces professionnels-enseignants, **lesquels sont impliqués** dans l’enseignement aux résidents en médecine familiale ? ***(Vous pouvez cocher plus d’une réponse).***
  ___ Infirmières cliniciennes
  ___ Travailleuses sociales
  ___ Psychologues
  ___ Pharmaciennes
  ___ Nutritionnistes
  ___ Infirmières praticiennes en soins de première ligne (IPSPL)
  ___ Physiothérapeutes
  ___ Kinésiologues
  ___ Professionnel de recherche
3. Dans votre milieu de résidence, **quelles sont les activités d’enseignement** qui relèvent (en partie ou complètement) des professionnels-enseignants? ***(Vous pouvez cocher plus d’une réponse).***
  ___ Enseignement formel (DCC, EMS, cours transversaux, GRef, thérapie de soutien, etc.)
  ___ Séminaires
  ___ Activités d’enseignement liées à la collaboration interprofessionnelle
  ___ Supervision directe
  ___ Supervision par discussion de cas
  ___ Observation du médecin de famille
  ___ Clubs de lecture
  ___ Rallye-ressources
  ___ Activités de recherche (Ex : EQEP)
  ___ Autre, précisez _________________________________________________________
4. Selon vous, y a-t-il d’autres activités d’enseignement pour lesquelles les professionnels-enseignants devraient être présents ?
  1. Oui, précisez : _____________________________________________________
  2. Non

### SECTION C : CONTRIBUTION DE L’ENSEIGNEMENT INTERDISCIPLINAIRE À MES APPRENTISSAGES

***En vous référant à l’ensemble des activités d’enseignement réalisées par les professionnels-enseignants de votre UMF, veuillez cocher, à l’aide de l’échelle suivante, votre degré d’accord avec chacun des énoncés.***

**Table:**

**1** **Pas du tout d’accord**	**2** **Un peu d’accord**	**3** **Moyennement d’accord**	**4** **Très en accord**	**5** **Totalement en accord**

1. De manière générale, l’enseignement interdisciplinaire…
  **Table:**
  *APPRENTISSAGES*						
  1.	Favorise l’apprentissage des gestes techniques	1	2	3	4	5
  2.	Favorise l’apprentissage/intégration des diagnostics différentiels	1	2	3	4	5
  3.	Me permet d’acquérir des nouvelles connaissances sur les problèmes de santé de mes patients	1	2	3	4	5
  4.	Favorise l’apprentissage de la thérapie de soutien	1	2	3	4	5
  5.	Favorise l’apprentissage d’un examen physique optimal	1	2	3	4	5
  6.	Favorise l’apprentissage d’un examen mental optimal	1	2	3	4	5
  7.	Favorise l’apprentissage de stratégies de communication avec les patients	1	2	3	4	5
  8.	Favorise le développement d’habiletés de négociation auprès des patients	1	2	3	4	5
  9.	Augmente mes connaissances sur les options disponibles pour traiter les problèmes de mes patients	1	2	3	4	5
  *COMPLÉMENTARITÉ*						
  10.	Complète les apprentissages effectués dans ma propre discipline	1	2	3	4	5
  11.	M’offre une perspective additionnelle afin de mieux cerner les problèmes de santé de mes patients	1	2	3	4	5
  12.	Favorise l’identification d’une plus grande diversité de stratégies cliniques auprès de mes patients (outils, prise en charge, etc.)	1	2	3	4	5
  *RÉFLEXIVITÉ*						
  13.	Me permet de réfléchir activement à la relation médecin-patient	1	2	3	4	5
  14.	Augmente mon niveau de confort face à des situations cliniques moins typiques et/ou plus complexes	1	2	3	4	5
  15.	Favorise le développement et l’intégration d’une pratique reflexive	1	2	3	4	5
  16.	Contribue à développer une plus grande confiance en mes compétences et habiletés	1	2	3	4	5
  17.	Me permet d’avoir une rétroaction bénéfique sur le développement de mes habiletés et connaissances en médecine familiale	1	2	3	4	5
  *COLLABORATION INTERPROFESSIONNELLE*						
  18.	Me permet de mieux cerner le rôle de l’ensemble des professionnels impliqués dans mon milieu de résidence	1	2	3	4	5
  19.	Favorise une perception positive des autres catégories de professionnels de la santé	1	2	3	4	5
  20.	Me prépare à effectuer des suivis conjoints avec d’autres professionnels de la santé	1	2	3	4	5
  21.	Me permet d’orienter efficacement les patients vers les diverses ressources disponibles dans le réseau de la santé	1	2	3	4	5
2. En vous référant aux énoncés de la question précédente, veuillez indiquer quels sont pour vous les 3 principaux bénéfices découlant de l’enseignement interdisciplinaire *(Inscrire le numéro correspondant à chacun de vos choix)*
  1^er^ choix _______ 2^ième^ choix _______ 3^ième^ choix ______
3. A quel moment avez-vous appris que des professionnels-enseignants allaient participer à votre évaluation ?
  1. Dès le début de la résidence
  2. Lors de ma toute première évaluation en médecine familiale
  3. Lors d’une évaluation ultérieure en médecine familiale
  4. J’ignorais que les professionnels-enseignants de mon UMF participaient à l’évaluation de mes blocs-stage en médecine familiale

### SECTION D : APRÉCIATION DE L’ENSEIGNEMENT INTERDISCIPLINAIRE

***Veuillez cocher, à l’aide de l’échelle suivante, votre degré d’accord avec chacun des énoncés.***

**Table:**

**1** **Pas du tout d’accord**	**2** **Un peu d’accord**	**3** **Moyennement d’accord**	**4** **Très en accord**	**5** **Totalement en accord**

- De manière générale, les professionnels-enseignants impliqués dans mon UMF…
  **Table:**
  **1.**	Sont impliqués de manière active face à l’enseignement aux résidents	1	2	3	4	5
  **2.**	Offrent une contribution pertinente à l’enseignement aux résidents	1	2	3	4	5
  **3.**	Transmettent des connaissances et des compétences qui me seront utiles dans ma pratique en tant que médecin de famille	1	2	3	4	5
  **4.**	Savent transmettre leur expertise en tenant compte de ma réalité de résident en médecine familiale	1	2	3	4	5
  **5.**	Font preuve de compétence dans leur rôle d’enseignant en médecine familiale	1	2	3	4	5
  **6.**	Font preuve d’engagement et de disponibilité dans leur rôle d’enseignant en médecine familiale	1	2	3	4	5
  **7.**	Offrent une contribution pertinente à l’évaluation des blocs-stages effectués en UMF	1	2	3	4	5
  **8.**	Favorisent le développement de mon autonomie professionnelle	1	2	3	4	5
  **9.**	Stimulent ma motivation en tant qu’apprenant	1	2	3	4	5
  **10.**	Exposent à une plus grande diversité de stratégies d’enseignement	1	2	3	4	5
  **11.**	Me préparent à ma future pratique en tant que médecin	1	2	3	4	5
- **Parmi les propositions suivantes, veuillez cocher les 2 conditions qui, selon vous, favorisent le plus votre intérêt et votre motivation en situation d’enseignement interdisciplinaire :**
  ___ Pertinence du contenu enseigné pour la médecine familiale
  ___ Motivation et dynamisme du professionnel-enseignant
  ___ Expertise du professionnel-enseignant quant au contenu présenté
  ___ Capacité du professionnel-enseignant à transmettre son expertise en l’adaptant à mes besoins en tant que résident en médecine familiale
  ___ Connaissance du cursus en médecine familiale
  ___ Présence d’un médecin de famille en tant que co-enseignant
- **Votre perception de l’enseignement interdisciplinaire a-t-elle évolué entre le début de votre résidence en médecine familiale et aujourd’hui?**
  1. Oui, favorablement
  2. Oui, défavorablement
  3. Non, ma perception initiale « favorable » est restée la même
  4. Non, ma perception initiale « défavorable » est restée la même
- **Voyez-vous un ou des aspects négatifs à l’enseignement interdisciplinaire en médecine familiale?**
  1. Oui, précisez ________________________________________________________
    ________________________________________________________
    ________________________________________________________
  2. Non

## Appendix B Interview Guide

### GUIDE D’ENTREVUE

#### INTRODUCTION

- Présentation du projet de recherche
- Présentation et signature du formulaire de consentement
- Définition du concept d’enseignement interdisciplinaire, qui se définit comme suit:
  o *« Un processus reposant sur l’utilisation et l’intégration de méthodes et de cadres d’analyse provenant de plus d’une discipline».* De manière spécifique, l’entrevue d’aujourd’hui portera sur l’ensemble des activités d’enseignement en UMF qui sont dispensées par des professionnels provenant d’autres disciplines que la médecine (ex : pharmacie, soins infirmiers, psychologie, service social, etc…).

1. **DESCRIPTION DE L’EXPÉRIENCE D’ENSEIGNEMENT INTERDISCIPLINAIRE**
  - Quels types de professionnels non-médecins sont présents dans votre milieu de résidence ?
  - Parmi ces professionnels, quels sont ceux qui participent à l’enseignement ?
  - Dans votre UMF, quelles sont les activités d’enseignement qui relèvent des professionnels-enseignants ?
2. **RÉACTIONS INITIALES**
  - À quel moment avez-vous appris que des professionnels non-médecins allaient vous enseigner au cours de votre résidence ?
    - De quelle manière avez-vous réagi ?
    - Aviez-vous des appréhensions ? Lesquelles ?
  - À quel moment avez-vous appris que des professionnels non-médecins contribueraient à votre évaluation en tant que résident en médecine familiale ?
    - De quelle manière avez-vous réagi ?
3. **ILLUSTRATIONS CONCRÈTES**
  - Décrivez une situation d’enseignement interdisciplinaire (cas avec un patient ou lors d’un cours) vécue au cours de votre résidence et que vous avez perçue comme utile.
    - De quel type d’enseignement s’agissait-il ?
    - Par quel type de professionnel non-médical cet enseignement a été effectué ?
    - Quelles ont été les retombées pour vous en tant que résident ?
    - Selon vous, auriez-vous fait les mêmes apprentissages si cette activité avait été enseignée uniquement par un médecin ? Qu’est-ce qui aurait été différent ?
  - Décrivez une situation d’enseignement multidisciplinaire vécue au cours de votre résidence et que vous avez perçue comme peu utile.
    - De quel type d’enseignement s’agissait-il ?
    - Par quel type de professionnel non-médical cet enseignement a été effectué ?
    - En quoi cet enseignement vous est-il apparu peu utile ?
    - Selon vous, auriez-vous fait davantage d’apprentissages si cette activité avait été enseignée uniquement par un médecin ? Qu’est-ce qui aurait été différent ?
4. **APPRÉCIATION**
  - Quelle est le principal bénéfice découlant de l’enseignement interdisciplinaire dans la résidence en médecine familiale ?
  - Selon vous, certaines des activités d’enseignement dispensées par des non-médecins devraient-elles plutôt être enseignées par des médecins ?
  - Vos attentes envers l’enseignement sont-elles les mêmes lorsqu’un médecin vous enseigne ?
  - Quelles conditions devraient être présentes pour qu’un résident en médecine familiale soit motivé à recevoir de l’enseignement par des non-médecins ?
  - Au terme de votre résidence, de quelle manière est-ce que votre perception de l’enseignement multidisciplinaire a évolué ?
5. **RECOMMANDATIONS**
  - Selon vous, de quelle manière les professionnels non-médecins devraient-ils être impliqués dans l’enseignement aux résidents en médecine de famille ?

#### CONCLUSION

Y a-t-il d’autres aspects qui n’ont pas été abordés au cours de notre entretien et dont vous aimeriez faire mention, afin de nous aider à cerner votre expérience d’apprentissage en contexte d’enseignement interdisciplinaire ?

**MERCI BEAUCOUP DE VOTRE PARTICIPATION**

## References

[1] Goldsmith AH, Hamilton D, Hornsby K, Wells D Interdisciplinary approaches to teaching, 2014 Available at: http://serc.carleton.edu/sp/library/interdisciplinary/index.html [Accessed February 9, 2017].

[2] The College of Family Physicians of Canada Specific Standards for Family Medicine Residency Programs Accredited by the College of Family Physicians of Canada, 2016 Available at: http://www.cfpc.ca/uploadedFiles/Red%20Book%20English.pdf [Accessed February 9, 2017].

[3] Jorgenson D, Muller A, Whelan AM, Buxton K Pharmacists teaching in family medicine residency programs. Can Family Physician. 2011;57(9):e341-6. PMC3173442 21918131

[4] Riesenberg LA, Little BW, Wright V Nonphysician medical educators: A literature review and job description resource. Acad Med. 2009;84(8):1078-88. 19638778 10.1097/ACM.0b013e3181ad1a05

[5] Lattuca L, Voight LJ, Fath KQ Does interdisciplinarity promote learning? Theoretical support and researchable questions. The Review of Higher Education. 2004;28(1):23-48.

[6] Solberg LI, Nesvacil LJ, Stoller JE Patient education by a family nurse as a model for training residents. Family Practice. 1989;6(2):114-9. 2501130 10.1093/fampra/6.2.114

[7] Liese BS, Shepherd DD, Cameron CL, Ojeleye AE Teaching psychological knowledge and skills to family physicians. Journal of Clinical Psychology in Medical Settings. 1995;2(1):21-38. 24225985 10.1007/BF01988625

[8] Mires GJ, Williams FLR, Harden RM, Howie PW, McCarey M, Robertson A Multiprofessional education in undergraduate curricula can work. Medical Teacher. 1999;21(3):281-5.

[9] Saultz J Interdisciplinary family medicine. Family Medicine. 2013;45(10):739-40. 24347197

[10] Bradley P, Bond V, Bradley P A questionnaire survey of students’ perceptions of nurse tutor teaching in a clinical skills learning programme. Medical Teacher. 2006;28(1):49-52. 16627324 10.1080/01421590500271332

[11] Brown C, Pollack A Reconstructing the paradigm: Teaching across the disciplines. J Undergrad Neurosci Educ. 2004;3(1):9-15. PMC3592600 23493654

[12] Carpenter J, Hewstone M Shared learning for doctors and social workers: Evaluation of a programme. British Journal of Social Work. 1996;26(2):239-57.

[13] Tarpley MJ, Davidson MA, Tarpley JL The role of the nonphysician educator in general surgery residency training: From outcome project and duty-hours restrictions to the next accreditation system and milestones. Journal of Surgical Education. 2014;71(1):119-24. 24411434 10.1016/j.jsurg.2013.06.004

[14] Hébert C Groupe de médecine de famille. Quelle aide peuvent apporter les autres professionnels de la santé? Le Médecin du Québec. 2016;51(2):10-4.

[15] Gouvernement du Québec Guides d’intégration des professionnels en GMF, 2015 [Internet]. Available at: http://publications.msss.gouv.qc.ca/msss/document-001529 [Accessed February 9, 2017].

[16] Gadsby K, Deighton C The perceptions of final year medical students in rheumatology workshops when delivered by a consultant and a nurse clinical educator. Rheumatology. 2005;44(11):1047-50. 15888501 10.1093/rheumatology/keh684

[17] Henley E, Glasser M, May J Medical student evaluation of family nurse practitioners as teachers. Family Medicine. 2000;32(7):491-4. 10916716

[18] Larue C, Loiselle CG, Bonin JP, Cohen R, Gélinas C, Dubois S. et al. Les méthodes mixtes : stratégies prometteuses pour l’évaluation des interventions infirmières. Recherche en soins infirmiers. 2009;97(2):50-62. 19642477

[19] Mayer R, Deslauriers JP Quelques éléments d’analyse qualitative In: Mayer R, Ouellet F, Saint-Jacques MC, Turcotte D, eds., Méthodes de recherche en intervention sociale. Montréal, Canada : Gaétan Morin Éditeur, 2000:159-89.

[20] Deslauriers JP Recherche qualitative : guide pratique. Montréal : McGraw-Hill, 1991.

